# Relationship between antidementia medication and fracture prevention in patients with Alzheimer’s dementia using a nationwide health insurance claims database

**DOI:** 10.1038/s41598-023-34173-0

**Published:** 2023-04-27

**Authors:** Tatsuya Hosoi, Mitsutaka Yakabe, Shoya Matsumoto, Kenji Fujimori, Junko Tamaki, Shinichi Nakatoh, Shigeyuki Ishii, Nobukazu Okimoto, Kuniyasu Kamiya, Masahiro Akishita, Masayuki Iki, Sumito Ogawa

**Affiliations:** 1 Department of Geriatric Medicine, Graduate School of Medicine, The University of Tokyo, 7-3-1 Hongo, Bunkyo-Ku, Tokyo, 113-8655 Japan; 2 Department of Health Administration and Policy, Tohoku University School of Medicine, 2-1 Seiryo-Machi, Aoba-Ku, Sendai, Miyagi 980-8575 Japan; 3 Department of Hygiene and Public Health, Faculty of Medicine, Osaka Medical and Pharmaceutical University, 2-7 Daigakumachi, Takatsuki, Osaka 569-8686 Japan; 4 Department of Orthopedic Surgery, Asahi General Hospital, 477 Tomari, Asahimachi, Shimo-Nikawa-Gun, Toyama, 939-0798 Japan; 5 Department of Regulatory Science, School of Pharmacy, Tokyo University of Pharmacy and Life Sciences, 1432-1 Horinouchi, Hachiouji, Tokyo 193-0392 Japan; 6 Okimoto Clinic, 185-4 Kubi, Yutaka-Machi, Kure, Hiroshima 734-0304 Japan; 7 Kindai University Faculty of Medicine, 377-2 Oono-Higashi, Osaka-Sayama, Osaka 589-8511 Japan; 8 National Database Japan-Osteoporosis Management (NDBJ-OS) Study Group, Kindai University Faculty of Medicine, 377-2 Oono-Higashi, Osaka-Sayama, Osaka 589-8511 Japan

**Keywords:** Medical research, Epidemiology

## Abstract

This retrospective study aimed to evaluate the association between antidementia medication use and incidence of new vertebral, hip, and radial fractures in patients with Alzheimer’s dementia (AD). We used the nationwide health insurance claims database of Japan from 2012 to 2019 and identified 12,167,938 patients aged ≥ 65 years who were newly registered from April 2012 to March 2016 and had verifiable data receipt from half-year before to 3 years after the registration. Among these patients, 304,658 were diagnosed with AD and we showed the prescription status of antidementia and osteoporosis medication among them. Propensity score matching was conducted for AD group with and without antidementia medication use, and 122,399 matched pairs were yielded. The incidence of hip fractures (4.0% vs. 1.9%, *p* < 0.001) and all clinical fractures (10.5% vs. 9.0%, *p* < 0.001) significantly decreased and that of radial fractures increased (0.6% vs. 1.0%, *p* < 0.001) in AD patients with antidementia medication use compared with AD patients without antidementia medication use. No significant difference was found in vertebral fractures (6.6% vs. 6.5%, *p* = 0.51). Overall, these results suggest a positive relationship between antidementia medication use and fracture prevention in patients with AD.

## Introduction

Given the rapidly increasing number of older people worldwide and parallel increase in patients with dementia, taking good care of them and extending “healthy life expectancy” are urgently required. Japan is one of the most aged countries worldwide, and the prevalence of dementia among older adults is reported to be 16.7% in 2020 (approximately 6.02 million people) and are expected to increase like in other East Asian countries^1,2^. Patients with dementia are likely to have lower dietary intake and suffer from malnutrition and weight loss^3,4^. Moreover, dementia is an independent risk factor for falls and bone fractures, leading to disability and need for nursing care^5,6^. Therefore, dementia has become a major health challenge globally, requiring a thoughtful and effective approach to its management. Alzheimer’s dementia (AD) is the most prevalent form of dementia, and approximately 70% of patients with dementia are diagnosed with AD, followed by vascular dementia, Lewy body dementia and Parkinson’s disease with dementia, and mixed dementia^4^. Acetylcholinesterase inhibitors (ChEIs), donepezil, galantamine, and rivastigmine are prescribed for patients with mild-to-moderate AD, whereas memantine, an *N*-methyl-d-aspartate (NMDA) receptor antagonist, is prescribed to patients with moderate-to-severe AD. These pharmacological treatments can alleviate AD symptoms; however, there is no cure for AD at the current moment, and clinical significance remains controversial^7–9^.

Physical function also declines with age, and frailty and sarcopenia cause falls and fractures. In older adults, 5–10% of falls resulted in fractures, and up to 90% of all fractures resulted from a fall^10^. Rates of bone fractures and dementia increase with age, and older adults with frailty had 1.4–3.6 times higher rates of dementia than normal older adults, and vice versa, suggesting a strong association between them^11^. A study also reported that dementia was significantly associated with a complete loss of walking ability after a hip fracture^12^. Therefore, early intervention for dementia may lead to fracture prevention.

The National Database of Health Insurance Claim Information and Specified Medical Checkups of Japan (NDBJ) is a large Japanese medical database that covers almost all claims in Japan since 2008. For this retrospective cohort study, we focused on patients with AD and aimed to clarify the current state of bone fractures and prescription of antidementia or osteoporosis medications, using this nationwide database. Our study answers the important questions of whether antidementia medication use was associated with fracture prevention.

## Results

**Figure 1:**
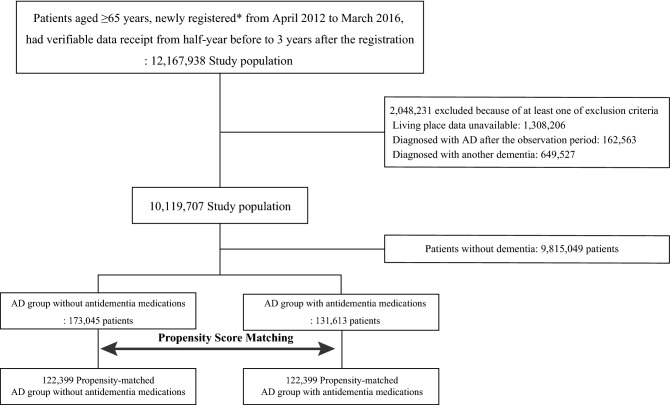
Flow diagram for the patient selection. Flowchart showing how cases and controls were selected from the nationwide health insurance claims database of Japan. *AD* Alzheimer’s dementia. *Newly registered as the first medical claim since turning 65 years old.

Figure 1 illustrates the patient selection process. We identified 12,167,938 patients aged ≥ 65 years who were newly registered from April 2012 to March 2016 and had verifiable data receipt from half-year before to 3 years after the registration. Among the identified patients, 2,048,231 were excluded because of unavailable background information, diagnosis of AD after the observation period, or diagnosis of another dementia type. Finally, 10,119,707 patients met the inclusion criteria (9,815,049 non-AD and 304,658 AD groups).

The baseline characteristics of the non-AD and AD groups are shown in Table 1. The AD group was older and included more female patients than the non-AD group. The rate of undergoing bone mineral density testing and the prevalence of osteoporosis were comparable between the groups, though the AD group was more frequently prescribed osteoporosis medications. Among patients diagnosed with osteoporosis, bisphosphonates (BP, 45.4%) were the most frequently prescribed osteoporosis medication before the registration, followed by vitamin D (alfacalcidol, 25.9%; eldecalcitrol, 14.3%), and selective estrogen receptor modulators (SERM, 15.5%).

**Table 1:** Baseline characteristics of the patients with and without AD.

Total, n	N=	Patients without AD		Patients with AD		Standardized difference
		9,815,049		304,658		
Age (years)	65–69	2,957,277	30.1%	13,763	4.5%	71.9
	70–74	3,421,484	34.9%	38,796	12.7%	53.8
	75–79	1,732,583	17.7%	55,542	18.2%	− 1.5
	80–84	1,023,049	10.4%	81,335	26.7%	− 42.8
	85–89	485,911	5.0%	73,611	24.2%	− 56.6
	90–94	154,994	1.6%	32,980	10.8%	− 39.1
	95–99	29,997	0.3%	6715	2.2%	− 17.1
	100+	9754	0.1%	1916	0.6%	− 8.8
Age mean (SD)		73.4	(73.43–73.44)	81.9	(81.84–81.89)	
Gender	Male	3,553,555	36.2%	73,572	24.1%	26.5
	Female	6,261,494	63.8%	231,086	75.9%	− 26.5
Osteoporosis		1,489,528	15.2%	43,368	14.2%	2.7
Bone fractures before entry	None	9,752,473	99.4%	298,161	97.9%	12.8
	Hip fracture	10,080	0.1%	1941	0.6%	− 8.8
	Vertebral fractures	52,043	0.5%	4470	1.5%	− 9.4
	Radius fracture	11	0.0%	0	0.0%	0.1
	Multiple fractures	442	0.0%	86	0.0%	− 1.9
Osteoporosis medications before entry	Bisphosphonate	667,641	6.8%	27,985	9.2%	− 8.8
	PTH	25,175	0.3%	2219	0.7%	− 6.7
	Dmab	687	0.0%	698	0.2%	− 6.5
	Eldecalcitrol	206,650	2.1%	12,293	4.0%	− 11.2
	Alfacalcidol	380,479	3.9%	17,154	5.6%	− 8.3
	SERM	227,813	2.3%	9931	3.3%	− 5.7
Bone mineral density testing	DEXA	170,864	1.7%	5837	1.9%	− 1.3
	MD	491,352	5.0%	15,980	5.2%	− 1.1
	US	92,818	0.9%	3238	1.1%	− 1.2

*AD* Alzheimer’s dementia, *SD* standard deviation.

The baseline characteristics of the AD group without antidementia medication and the AD group with antidementia medication before and after PS matching are shown in Table 2. PS matching yielded 122,399 pairs, and the C-statistic for the logistic regression was 0.59. In Table 2, although the mean age was almost the same between the groups, by age group, the prevalence of antidementia medication use was markedly higher in the AD group aged 75–84 (51.7%), and it became lower as patients aged (85–89 years, 44.4%; 90–94 years, 31.6%; 95–99 years, 22.1%; ≥ 100 years, 3.5%). In total, 43.2% were prescribed at least one type of antidementia medications, and the most prescribed medication was donepezil (74.9%), followed by memantine (33.2%), galantamine (21.9%), and rivastigmine patch (2.9%). Moreover, the AD group prescribed antidementia medications more frequently underwent bone mineral density testing and prescribed osteoporosis medications, although the prevalence of osteoporosis was not much different. After PS matching, the standardized differences were all < 10%, indicating well-balanced distributions of the patient characteristics.

**Table 2:** Baseline characteristics of AD group with and without antidementia medications.

Total, n	N=	Without antidemenita medications (before PS matching)		With antidemenita medications (before PS matching)		Standardized difference	Without antidemenita medications (after PS matching)		With antidemenita medications (after PS matching)		Standardized difference
		173,045		131,613			122,399		122,399		
Age (years)	65–69	9340	5.4%	4423	3.4%	10.0	6288	5.1%	4318	3.5%	7.9
	70–74	26,500	15.3%	12,296	9.3%	18.2	14,796	12.1%	11,814	9.7%	7.8
	75–79	27,340	15.8%	28,202	21.4%	− 14.5	23,928	19.5%	26,467	21.6%	− 5.1
	80–84	39,299	22.7%	42,036	31.9%	− 20.8	34,310	28.0%	38,542	31.5%	− 7.6
	85–89	40,920	23.6%	32,691	24.8%	− 2.8	29,555	24.1%	29,974	24.5%	− 0.8
	90–94	22,570	13.0%	10,410	7.9%	16.8	11,199	9.1%	9,786	8.0%	4.1
	95–99	5,228	3.0%	1,487	1.1%	13.3	1,999	1.6%	1,430	1.2%	4.0
	100 +	1,848	1.1%	68	0.1%	13.7	324	0.3%	68	0.1%	5.2
Age mean (SD)		82.0	(81.96–82.03)	81.7	(81.66–81.73)		81.4	(81.40–81.47)	81.6	(81.59–81.66)	
Gender	Male	39,633	22.9%	33,939	25.8%	− 6.7	34,220	28.0%	33,378	27.3%	1.5
	Female										
Antdementia medications use	Donepezil	–	–	98,572	74.9%	–	–	–	91,500	74.8%	–
	Galantamine	–	–	28,882	21.9%	–	–	–	26,828	21.9%	–
	Rivastigmine patch	–	–	3881	2.9%	–	–	–	3593	2.9%	–
	Memantine	–	–	43,661	33.2%	–	–	–	40,854	33.4%	–
Bone fractures before entry	None	169,445	97.9%	128,716	97.8%	0.8	120,356	98.3%	119,927	98.0%	2.6
	Hip fracture	1293	0.7%	648	0.5%	3.2	575	0.5%	591	0.5%	− 0.2
	Vertebral fractures	2253	1.3%	2217	1.7%	− 3.2	1440	1.2%	1854	1.5%	− 2.9
	Radius fracture	0	0.0%	0	0.0%	–	0	0.0%	0	0.0%	–
	Multiple fractures	54	0.0%	32	0.0%	0.4	28	0.0%	27	0.0%	0.1
Osteoporosis		26,014	15.0%	17,354	13.2%	5.3	17,898	14.6%	16,570	13.5%	3.1
Osteoporosis medications before entry	Bisphosphonate	11,208	6.5%	16,777	12.7%	− 21.4	11,139	9.1%	10,854	8.9%	0.8
	PTH	762	0.4%	1457	1.1%	− 7.6	759	0.6%	806	0.7%	− 0.5
	Dmab	21	0.0%	677	0.5%	− 9.8	21	0.0%	20	0.0%	0.1
	Eldecalcitrol	3590	2.1%	8703	6.6%	− 22.4	3590	2.9%	3217	2.6%	1.9
	Alfacalcidol	8108	4.7%	9046	6.9%	− 9.4	7360	6.0%	6835	5.6%	1.8
	SERM	4250	2.5%	5681	4.3%	− 10.3	4075	3.3%	4501	3.7%	− 1.9
Bone mineral density testing	DEXA	2386	1.4%	3451	2.6%	− 8.9	2117	1.7%	2170	1.8%	− 0.3
	MD	6902	4.0%	9078	6.9%	− 12.8	6264	5.1%	5822	4.8%	1.7
	US	1393	0.8%	1845	1.4%	− 5.7	1257	1.0%	1255	1.0%	0.0

*AD* Alzheimer’s dementia, *SD* standard deviation.

The main results are shown in Table 3, and the incidence of hip fractures (4.0% vs. 1.9%; risk difference, − 2.1% [95% confidence interval (CI) − 2.2 to − 2.0%]; number needed to treat (NNT) 47, [95% CI 45–50]) and all clinical fractures (10.5% vs. 9.0%; risk difference, − 1.5% [95% CI − 1.8 to − 1.3%]; NNT 65, [95% CI 57–77]) significantly decreased, whereas that of radial fractures increased (0.6% vs. 1.0%, *p* < 0.001) in the AD group with antidementia medication compared with the AD group without antidementia medication. No significant difference was found in vertebral fractures (6.6% vs. 6.5%, *p* = 0.51). To mitigate the impact of competing risks, we also presented the results as cases per 100 person-years, which showed the similar results.

**Table 3:** Outcomes in the AD group with and without antidementia medications after propensity score matching.

Group	Outcome	Without antidementia medications	With antidementia medications	Risk difference	
				(95% CI)	*p-*value
All patients (122,399 pairs)	Hip fracture, *n* (%)	4877 (4.0) 1.33/100 person-year	2294 (1.9) 0.63/100 person-year	− 2.1% (− 2.2 to − 2.0)	< 0.001
	Vertebral fracture, n (%)	8045 (6.6) 2.27/100 person-year	7965 (6.5) 2.24/100 person-year	− 0.1% (− 0.3 to 0.1)	0.51
	Radius fracture, n (%)	717 (0.6) 0.20/100 person-year	1177 (1.0) 0.32/100 person-year	0.4% (0.3–0.4)	< 0.001
	All clinical fractures, n (%)	12,875 (10.5) 3.52 /100 person-year	10,998 (9.0) 3.00/100 person-year	− 1.5% (− 1.8 to − 1.3)	< 0.001

*AD* Alzheimer’s dementia, *CI* confidence interval.

Supplementary Table 1 shows the baseline characteristics of the three AD groups with antidementia medications: AD group with ChEI as a monotherapy (including patients with prescription changes), with memantine as a monotherapy, and with both ChEI and memantine as multitherapy. Multitherapy group was younger than monotherapy group (mean age (SD); 80.8 (6.1) vs. 82.1 (6.1)) and the prevalence of galantamine and rivastigmine patch was higher. Supplementary Fig. 1 illustrates the PS matching in the subgroup analyses. The AD group without antidementia medication was compared with the AD groups with ChEI, with memantine, and with both ChEI + memantine. The C-statistics for the logistic regression were 0.59, 0.59, and 0.60, respectively. Patients’ backgrounds before and after PS matching are shown in Supplementary Tables 2–4, and the distributions of patient background variables were well balanced after PS matching. In Supplementary Tables 1–4, the number of patients included was < 10 in some rows and we combined them with other rows in order to guarantee the anonymity of the patients. Supplementary Table 5 shows the results of the subgroup analyses, and the incidence of hip fractures (ChEI, 4.1% vs. 1.6%, *p* < 0.001; memantine, 4.0% vs. 3.0%, *p* < 0.001; multitherapy, 3.7% vs. 2.2%, *p* < 0.001) and all clinical fractures (ChEI, 11.1% vs. 9.1%, *p* < 0.001; memantine, 11.0% vs. 9.6%, *p* < 0.001; multitherapy, 10.7% vs. 9.5%, *p* < 0.001) significantly decreased, and that of radial fractures increased (ChEI, 0.6% vs. 1.0%, *p* < 0.001; memantine, 0.5% vs. 0.9%, *p* = 0.001; multitherapy, 0.6% vs. 1.0%, *p* < 0.001) in each therapy group. No significant difference was found in the incidence of vertebral fractures, except for the memantine group (7.1% vs. 6.2%, *p* = 0.006).

## Discussion

In Japan, all residents are covered with public health insurance and recorded in NDBJ. Therefore, studies based on NDBJ enable us to better investigate the state of national medical care without regional and selected institution bias. Using this real-world database, we shed light on the association between antidementia medication use and incidence of bone fractures among patients with AD aged ≥ 65 years. Specifically, we determined the number of patients with AD who were prescribed antidementia medications, identified the patterns of antidementia or osteoporosis medication use, and investigated the fracture rates. PS-matched analyses showed that antidementia medication use was associated with decreased incidence of hip fractures and increased incidence of radial fractures. In total, the proportion of patients with clinical fractures at any location was significantly low among patients with AD who were using antidementia medications.

We identified 304,658 patients with AD, 76% of whom were female. During the study period, 43.2% of the patients with AD were prescribed at least one type of antidementia medication, and the most prescribed medication was donepezil, which was the ChEI approved for AD treatment and the most prescribed drug for dementia worldwide^13^. Memantine exhibited efficacy and safety in patients with moderate-to-severe AD and was mostly prescribed in combination with ChEIs^14^. By age groups, the prevalence of antidementia medication use was the highest in patients with AD aged 80–84 years (51.7%) and it became lower as they aged. Antidementia medications are associated with slower cognitive and functional ability decline, but the clinical significance remains unclear, especially when patients had severe AD or aged ≥ 85 years^7,15,16^. Old patients tend to suffer from polypharmacy and risks of drug–drug interactions, and clinical trials of antidementia medications have mainly focused on patients with dementia aged < 85 years; therefore, physicians might have avoided prescribing medications in very old individuals^7^.

Individuals who fractured their hip would suffer from subsequent hip fractures, and nearly 40% of them would be institutionalized or unable to walk independently within the year, 60% would require assistance a year later, and approximately one in four would die within a year^17–19^. Therefore, early prevention efforts for falls and fractures are needed, and this study pointed out the possibility that antidementia medication use was significantly associated with a decreased incidence of hip fractures or clinical fractures. Comparative analysis of fracture rates after PS matching indicated that antidementia medication use suppressed the incidence of hip fractures and all clinical fractures by 2.1% and 1.5%, respectively, and increased the incidence of radial fractures by 0.4%. We also conducted subgroup analyses in the monotherapy group (ChEI or memantine) and multitherapy group (ChEI + memantine) and confirmed the similar results in all medication types. Both hip and radial fractures are common fragility fractures resulting from falls; however, hip fractures can be prevented at the sacrifice of radial fractures in patients with good physical or cognitive function because they can land on their hands to protect themselves during a fall^20^. Therefore, a possible explanation for the present results was that antidementia medications prevented bruised hip joints in patients with AD by improving cognitive and physical functions. Since radius fractures have a lower incidence and less impact on ADLs compared with hip fractures, antidementia medications might have a generally positive effect on fractures. Early use of antidementia medications was reported to reduce the risk of admission to 24-h care, and this positive effect of antidementia medications on fractures, which we had reported, might have contributed to the reduction of institutionalization^21^.

Surprisingly, the rate of vertebral fractures decreased by 0.9% in the memantine group. Vertebral fractures are usually the first to occur in osteoporosis, provide indisputable evidence of reduced bone strength, and are frequently a harbinger of further vertebral and nonvertebral fracture^22^. In in vitro and animal experiments, the downregulation of NMDA receptor expression decreased osteogenesis^23^. On the contrary, a meta-analysis reported that memantine might have a favorable effect on fractures, with no effects on other events, such as syncope, falls, or accidental injuries^24,25^. Our results were inconsistent with those of animal studies but in line with real-world data, though the casual reason for this was unclear.

This study has several limitations. First, this is a retrospective study using data from a database, and the causal relationship between antidementia medication use and reduced incidence of new fractures cannot be assessed. Second, although the NDB contains exhaustive data, including information on nearly all health insurance claims, validation is a common issue worldwide when using administrative claims databases^26^. Although the diagnostic criteria for AD are relatively well-established, there is still a high rate of misdiagnoses and possible overdiagnosis of AD^13^. Third, this is a retrospective observational study, and confounding biases may be introduced. There are many clinical conditions, diseases, and medications that affect falls (e.g., sleeping pills, comorbidities, AD severity, and activity of daily living) and we could not fully consider the possible effects of these on the fracture risk. Although we used PS matching to adjust for numerous measured confounders, PS matching only accounts for the observed covariates, so residual confounding (e.g., socioeconomic factors, healthcare access or physician prescribing patterns) is possible. Forth, patients who died during the 3.5-year observation period were not included because of the limitation of the data and there was the risk of competing bias. A model that takes into account competing risks and censoring should have been constructed in order to explicitly state the association between AD drugs and the outcome.

## Conclusions

To the best of our knowledge, this is the first study suggesting that antidementia medication use was associated with a decreased incidence of hip fractures and all clinical fractures and increased risk of radial fractures. Memantine, an NMDA receptor antagonist, was associated with the decreased rate of vertebral fractures, indicating the difference from ChEIs in the mechanism of action. Further studies considering dementia progression and treatment change over time, dementia severity, activities of daily living, risk of falls, and the occurrence of death/termination events are needed^27^.

## Methods

### Study population and data sources

The NDBJ database has accumulated all monthly electronic health insurance claims and yearly specific health data on each patient, the details of which were described elsewhere^28^. Briefly, the data analyzed in this study were as follows: patient’s identification number; age and sex; region of residence; date of consultation for outpatient service and diagnosis; main diagnosis and comorbidities written in a code used in electronic receipt processing system; and date of procedures and drugs provided to each patient. We used 2 types of identifiers (ID 1 and 2, both 64 digits) to link the insurance claims of individual patients, collate the names, and construct the database. This allowed us to trace patients' information even when their ID changed over time^29,30^.

This study used NDBJ data from fiscal years (FY) 2012 to 2018 (April 1, 2012, to March 31, 2019). Among patients who were newly registered as the first medical claim from April 2012 to March 2016 since turning 65 years old, we identified those met the inclusion and exclusion criteria.

The inclusion criteria were as follows: (1) aged ≥ 65 years old at the date of entry, (2) had a look-back period of 6 months and a follow-up period of 3 years from the date of registration, (3) had verifiable data receipt throughout the 3.5-year observation period, (4) had never been prescribed antidementia medications in the 6 months before the date of entry. The verifiable data included the diagnosis of dementia (unrelated to AD medications), osteoporosis, and bone fractures, and the prescription of antidementia or osteoporosis medications, the codes used are provided in the supplementary information file. Each drug prescription is assigned a 9-digit code and each disease diagnosis is assigned a 7-digit Japanese Standard Disease Code. As for AD patients with antidementia medication use, the entry date was set at the day of prescribing antidementia medications for the first time. As for patients without dementia (not only AD) and AD patients without antidementia medication use, the entry date was set at the newly registered timing. The observation period for each patient was set at 3 years and no one died during the observation period. The design diagram that depicts these temporal anchors are shown in Supplementary Fig. 2.

We excluded patients who met any of the following exclusion criteria: (1) those who were prescribed antidementia medications but discontinued them for more than 30 days during the observation period, (2) those who were prescribed for more than 90 days at one time (because the maximum number of drugs that can be prescribed once is 90 in Japan), (3) those who were diagnosed with dementia after the observation period, (4) those who were prescribed medications that affect bone fractures (i.e., steroid, antidiabetic, antipodagric, and hormone medications).

### Data collection

Based on the data receipt, patient baseline characteristics included age, sex, region of residence, presence of osteoporosis, history of bone mineral density testing and bone fractures before entry, and prescription of antidementia medications and osteoporosis medications. For bone mineral density testing, we determined whether the test performed was a lumbar spine scan using dual-energy X-ray absorptiometry, micro densitometry, or ultrasonography. History of bone fractures was categorized into five groups: none, hip fractures, vertebral fractures, radial fractures, and multiple fractures. For osteoporosis medications, we chose the medications evaluated as “A” for the effect of vertebral fracture depression: BP (both oral and injectable BPs), parathyroid hormone (PTH, both daily and weekly teriparatide), anti-receptor activator of nuclear factor kappa-Β ligand antibodies, active vitamin D3 single-agent (eldecalcitol) and others (alfacalcidol and menatetrenone), and SERM^31^.

### Outcomes

We divided patients into the non-AD group, AD group without antidementia medication use, and AD group with antidementia medication use. The primary outcome was the rates of hip, vertebral, radial, and all clinical fractures during the observation period. When multiple fractures were registered, we extracted the data of the first fracture registered. Patients with ≥ 2 fractures registered on the same day were assigned to each fracture.

In the main analysis, we compared the fracture rates between the AD group with and without antidementia medication use. In the subgroup analyses, we divided patients in the AD group with antidementia medication use into three groups: AD group with ChEI as a monotherapy (including patients with prescription changes), group with memantine as monotherapy, and group with multitherapy (both ChEI and memantine).

### Statistical analyses

First, we showed the background characteristics of each group. In the main analysis, we conducted one-to-one PS matching between the AD group with and without antidementia medication use^32–34^. For PS estimation, we used a logistic regression model with antidementia medication use as the function for patient background characteristics. The C-statistic was calculated to evaluate the discriminatory ability of the model. By using PS estimates, we conducted nearest-neighbor matching without replacement, and the caliper was set at 0.2 times the standard deviation of the PS estimates^34^. Standardized differences were used to compare characteristics between the two groups before and after matching, and standardized differences of > 10% were regarded as imbalanced^35^. Outcomes were compared between the PS-matched patients in the AD group with and without antidementia medication use. In the subgroup analyses, we also conducted PS matching and compared the AD group without antidementia medication use and the three groups: ChEI group, memantine group, and multitherapy group.

We presented the numbers and percentages for categorical variables and means and standard deviations (or medians and interquartile ranges (IQRs)) for continuous variables. The Pearson χ^2^ test was used for categorical variables, with two-sided, and significance was defined as *p* < 0.05. All statistical analyses were conducted in Stata/SE version 17.0 (StataCorp, College Station, TX, USA).

### Ethics approval and consent to participate

The study protocol was approved by the Ministry of Health, Labour and Welfare, as well as the Institutional Review Board of the Graduate School of Medicine, The University of Tokyo (Approval number: 2020291NI). All methods were performed in accordance with the relevant guidelines and regulations. The data in this study were completely anonymous; thus, the need of informed consent was waived by the Institutional Review Board of the Graduate School of Medicine, The University of Tokyo. The present study protocol was approved by the Ethics Committee of the University of Tokyo Hospital.

## Supplementary Information

Supplementary Figure 1.

Supplementary Figure 2.

Supplementary Information.

Supplementary Tables.

## Data Availability

The dataset analyzed in the current study is not publicly available because of contracts with the hospitals providing data to the database.

## Code availability

The scripts detailing the analyses conducted are available at supplementary information file.

## References

[1] Cabinet Office. Annual Report on the Ageing Society (Cabinet Office, 2019). https://www8.cao.go.jp/kourei/whitepaper/w-2017/zenbun/29pdf_index.html. Accessed on 1 July 2017.

[2] Prince M (2016). Recent global trends in the prevalence and incidence of dementia, and survival with dementia. Alzheimers Res. Ther..

[3] Kalantar-Zadeh KK (2007). Risk factor paradox in wasting diseases. Curr. Opin. Clin. Nutr. Metab. Care.

[4] Herke M (2018). Environmental and behavioural modifications for improving food and fluid intake in people with dementia. Cochrane Database Syst. Rev..

[5] Tinetti ME, Speechley M, Ginter SF (1988). Risk factors for falls among elderly persons living in the community. N. Engl. J. Med..

[6] Bohlken J, Jacob L, Schaum P, Rapp MA, Kostev K (2017). Hip fracture risk in patients with dementia in German primary care practices. Dementia (London).

[7] Buckley JS, Salpeter SR (2015). A risk-benefit assessment of dementia medications: Systematic review of the evidence. Drugs Aging.

[8] Birks J, Harvey RJ (2006). Donepezil for dementia due to Alzheimer’s disease. Cochrane Database Syst. Rev..

[9] Ruangritchankul S, Chantharit P, Srisuma S, Gray LC (2021). Adverse drug reactions of acetylcholinesterase inhibitors in older people living with dementia: A comprehensive literature review. Ther. Clin. Risk Manag..

[10] Peeters G, van Schoor NM, Lips P (2009). Fall risk: The clinical relevance of falls and how to integrate fall risk with fracture risk. Best Pract. Res. Clin. Rheumatol..

[11] Robertson DA, Savva GM, Kenny RA (2013). Frailty and cognitive impairment—A review of the evidence. Ageing Res. Rev..

[12] Martinez-Carranza N, Lindqvist K, Modig K, Hedström M (2022). Factors associated with non-walking 4 months after hip fracture. A prospective study of 23,759 fractures. Injury.

[13] Cacabelos R (2022). Personalized management and treatment of Alzheimer’s disease. Life (Basel)..

[14] Tariot PN (2004). Memantine treatment in patients with moderate to severe Alzheimer disease already receiving donepezil: A randomized controlled trial. JAMA.

[15] Okumura Y, Sakata N (2018). Antidementia drug use in Japan: Bridging the research-to-practice gap. Int. J. Geriatr. Psychiatry.

[16] Rountree SD, Atri A, Lopez OL, Doody RS (2013). Effectiveness of antidementia drugs in delaying Alzheimer’s disease progression. Alzheimers Dement..

[17] Schemitsch E (2022). Hip fracture predicts subsequent hip fracture: A retrospective observational study to support a call to early hip fracture prevention efforts in post-fracture patients. Osteoporos. Int..

[18] Magaziner J, Simonsick EM, Kashner TM, Hebel JR, Kenzora JE (1990). Predictors of functional recovery one year following hospital discharge for hip fracture: A prospective study. J. Gerontol..

[19] Cooper C, Atkinson EJ, Jacobsen SJ, O’Fallon WM, Melton LJ (1993). Population-based study of survival after osteoporotic fractures. Am. J. Epidemiol..

[20] Soerensen S (2022). Epidemiology of distal forearm fracture: A population-based study of 5426 fractures. Hand (N Y)..

[21] Halminen O (2021). Early start of anti-dementia medication delays transition to 24-hour care in Alzheimer’s disease patients: A Finnish nationwide cohort study. J. Alzheimers Dis..

[22] Griffith JF, Guglielmi G (2010). Vertebral fracture. Radiol. Clin. N. Am..

[23] Ho ML (2005). Down-regulation of *N*-methyl d-aspartate receptor in rat-modeled disuse osteopenia. Osteoporos. Int..

[24] Hartikainen S, Bell JS (2011). Review: In people with dementia, cholinesterase inhibitors may increase syncope and memantine may reduce fractures. Evid. Based Ment. Health.

[25] Kim DH, Brown RT, Ding EL, Kiel DP, Berry SD (2011). Dementia medications and risk of falls, syncope, and related adverse events meta-analysis of randomized controlled trials. J. Am. Geriatr. Soc..

[26] Nakayama T (2017). Analysis of the evidence-practice gap to facilitate proper medical care for the elderly: Investigation, using databases, of utilization measures for National Database of Health Insurance Claims and Specific Health Checkups of Japan (NDB). Environ. Health Prev. Med..

[27] Hernán, M.A., Robins, J.M. Causal inference: What if. (Chapman & Hall/CRC, 2020). https://www.hsph.harvard.edu/miguel-hernan/causal-inference-book/. Accessed on 1 March 2023.

[28] Matsuda S, Fujimori K (2014). The claim database in Japan. Asian Pac. J. Dis. Manag..

[29] Kubo S, *et al*. National Database of Health Insurance Claims and Specific Health Checkups of Japan (NDB): Outline and Patient-Matching Technique. *bioRxiv*. Published online April 2, 2018, 280008. 10.1101/280008.

[30] Hashimoto Y (2022). Incidence of sympathetic ophthalmia after inciting events: A national database study in Japan. Ophthalmology.

[31] Japan Osteoporosis Society. Guidelines on the prevention and treatment of osteoporosis 2015. The committee for development of the guidelines on the prevention and treatment of osteoporosis. (in Japanese), 2015. https://www.josteo.com/ja/guideline/doc/15_1.pdf. Accessed on 1 July 2015.

[32] Austin PC (2011). Comparing paired vs non-paired statistical methods of analyses when making inferences about absolute risk reductions in propensity-score matched samples. Stat. Med..

[33] Jupiter DC (2017). Propensity score matching: Retrospective randomization?. J. Foot Ankle Surg..

[34] Hosoi T (2020). Association between comprehensive geriatric assessment and short-term outcomes among older adult patients with stroke: A nationwide retrospective cohort study using propensity score and instrumental variable methods. EClinicalmedicine.

[35] Austin PC (2009). Using the standardized difference to compare the prevalence of a binary variable between two groups in observational research. Commun. Stat. Simul. Comput..

