# Supplementary figures and images for: Relationship between antidementia medication and fracture prevention in patients with Alzheimer’s dementia using a nationwide health insurance claims database
Source: Sci Rep. 2023 Apr 27;13:6893. doi: 10.1038/s41598-023-34173-0 (PMC10140048; doi:10.1038/s41598-023-34173-0)

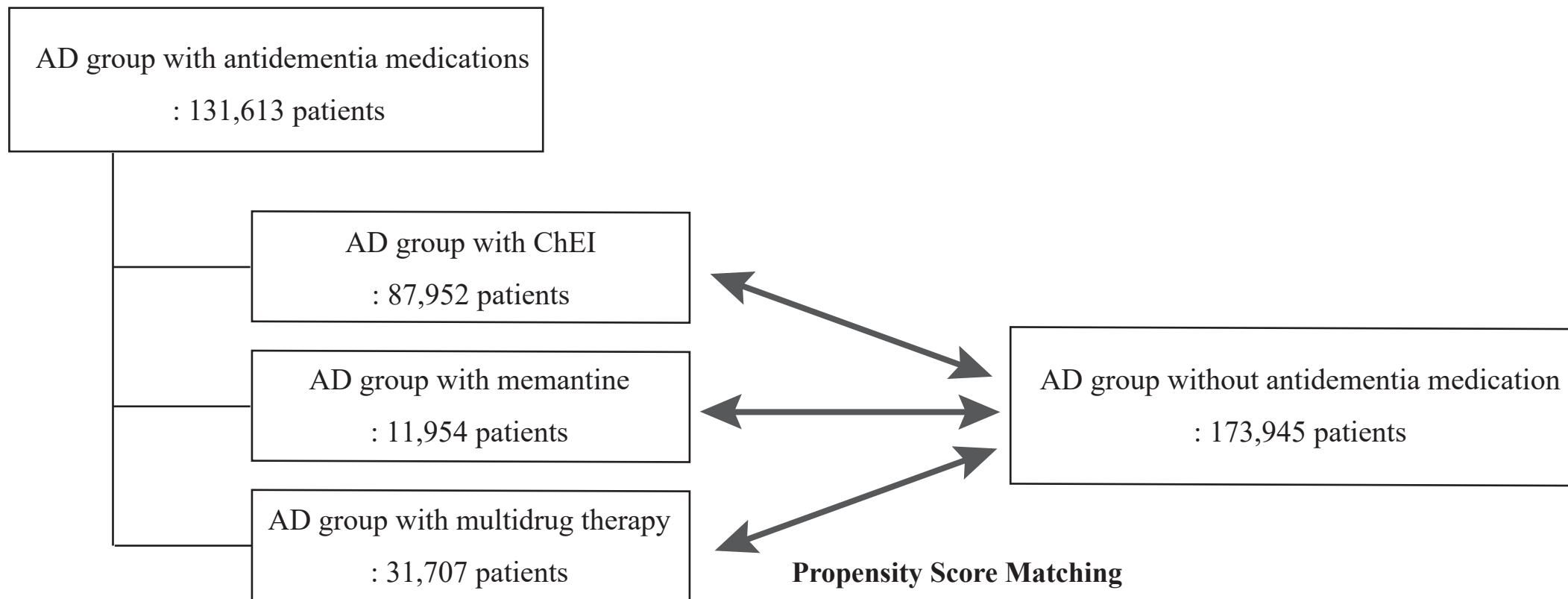

Supplement: Supplementary file 1 — Supplementary Figure 1. [file 41598_2023_34173_MOESM1_ESM.pdf]

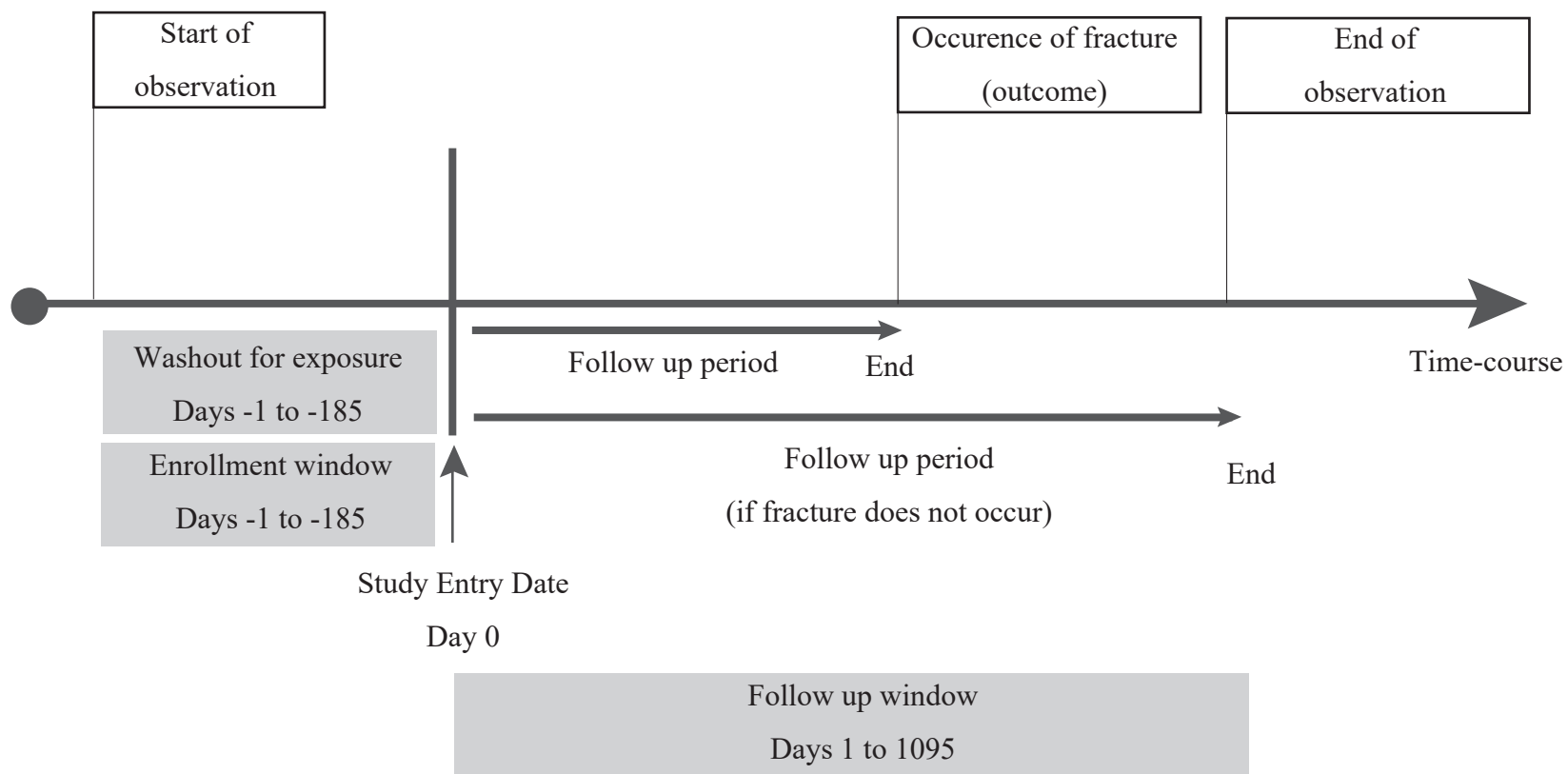

Supplement: Supplementary file 2 — Supplementary Figure 2. [file 41598_2023_34173_MOESM2_ESM.pdf]
