# Supplementary material for: Relationship between antidementia medication and fracture prevention in patients with Alzheimer’s dementia using a nationwide health insurance claims database
Source: Sci Rep. 2023 Apr 27;13:6893. doi: 10.1038/s41598-023-34173-0 (PMC10140048; doi:10.1038/s41598-023-34173-0)
Supplement: Supplementary file 4 — Supplementary Tables. [file 41598_2023_34173_MOESM4_ESM.docx]

**Supplementary Table 1. Baseline characteristics of AD group patients with ChEI, memantine, and multitherapy.**

|  |  | ChEI as a monotherapy | | Memantine as a monotherapy | | Multidrug therapy  (both ChEI and memantine) | |
| --- | --- | --- | --- | --- | --- | --- | --- |
| Total, n | N= | 87952 |  | 11,954 |  | 31,707 |  |
| Age (years) |  |  |  |  |  |  |  |
|  | 65-69 | 2,659 | 3.0% | 378 | 3.2% | 1,386 | 4.4% |
|  | 70-74 | 7,775 | 8.8% | 952 | 8.0% | 3,569 | 11.3% |
|  | 75-79 | 18,442 | 21.0% | 2,157 | 18.0% | 7,603 | 24.0% |
|  | 80-84 | 28,423 | 32.3% | 3,388 | 28.3% | 10,225 | 32.2% |
|  | 85-89 | 22,546 | 25.6% | 3,266 | 27.3% | 6,879 | 21.7% |
|  | 90-94 | 7,123 | 8.1% | 1,486 | 12.4% | 1,801 | 5.7% |
|  | 95+ | 984 | 1.1% | 327 | 2.7% | 244 | 0.8% |
| Age mean (SD) | | 81.9 | (81.83-81.91) | 82.9 | (82.74-82.98) | 80.8 | (80.71-80.85) |
| Gender | |  |  |  |  |  |  |
|  | male | 22,094 | 25.1% | 3,227 | 27.0% | 8,618 | 27.2% |
|  | female | 65,858 | 74.9% | 8,727 | 73.0% | 23,089 | 72.8% |
| Antdementia medications use | |  |  |  |  |  |  |
|  | Donepezil | 73,764 | 83.9% | - | - | 24,808 | 78.2% |
|  | Galantamine | 18,779 | 21.4% | - | - | 10,103 | 31.9% |
|  | Rivastigmine patch | 1,669 | 1.9% | - | - | 2,212 | 7.0% |
|  | Memantine | - | - | 11,954 | 100% | 31,707 | 100% |
| Bone fractures before entry | |  |  |  |  |  |  |
|  | None | 86,055 | 97.8% | 11,604 | 97.1% | 31,057 | 97.9% |
| Osteoporosis |  | 11,464 | 13.0% | 1,425 | 11.9% | 4,465 | 14.1% |
| Osteoporosis medications before entry | |  |  |  |  |  |  |
|  | Bisphosphonate | 11,634 | 13.2% | 1,329 | 11.1% | 3,814 | 12.0% |
|  | PTH | 1,023 | 1.2% | 128 | 1.1% | 306 | 1.0% |
|  | Dmab | 466 | 0.5% | 62 | 0.5% | 149 | 0.5% |
|  | Eldecalcitrol | 5,991 | 6.8% | 662 | 5.5% | 2,050 | 6.5% |
|  | Alfacalcidol | 6,386 | 7.3% | 748 | 6.3% | 1,912 | 6.0% |
|  | SERM | 3,943 | 4.5% | 456 | 3.8% | 1,282 | 4.0% |
| Bone mineral density testing | |  |  |  |  |  |  |
|  | DEXA | 2,383 | 2.7% | 269 | 2.3% | 799 | 2.5% |
|  | MD | 6,395 | 7.3% | 641 | 5.4% | 2,042 | 6.4% |
|  | US | 1,251 | 1.4% | 156 | 1.3% | 438 | 1.4% |

AD, Alzheimer’s dementia; ChEI, Acetylcholinesterase inhibitors; SD, standard deviation.

**Supplementary Table 2. Baseline characteristics of AD group with ChEI and without antidementia medications.**

|  |  | Without antidemenita medications (before PS matching) | | With ChEI as a monotherapy (before PS matching) | | Standardized Difference | Without antidemenita medications (after PS matching) | | With ChEI as a monotherapy (after PS matching) | | Standardized Difference |
| --- | --- | --- | --- | --- | --- | --- | --- | --- | --- | --- | --- |
| Total, n | N= | 173,045 |  | 87,952 |  |  | 85,192 |  | 85,192 |  |  |
| Age (years) | |  |  |  |  |  |  |  |  |  |  |
|  | 65-69 | 9,340 | 5.4% | 2,659 | 3.0% | 11.8 | 2,933 | 3.4% | 2,619 | 3.1% | 2.1 |
|  | 70-74 | 26,500 | 15.3% | 7,775 | 8.8% | 20.0 | 8,733 | 10.3% | 7,589 | 8.9% | 4.6 |
|  | 75-79 | 27,340 | 15.8% | 18,442 | 21.0% | -13.4 | 17,783 | 20.9% | 17,837 | 20.9% | -0.2 |
|  | 80-84 | 39,299 | 22.7% | 28,423 | 32.3% | -21.6 | 25,770 | 30.2% | 27,361 | 32.1% | -4.0 |
|  | 85-89 | 40,920 | 23.6% | 22,546 | 25.6% | -4.6 | 21,060 | 24.7% | 21,854 | 25.7% | -2.1 |
|  | 90-94 | 22,570 | 13.0% | 7,123 | 8.1% | 16.1 | 7,500 | 8.8% | 6,963 | 8.2% | 2.3 |
|  | 95+ | 7,076 | 4.1% | 984 | 1.1% | 18.7 | 1,413 | 1.7% | 969 | 1.1% | 4.4 |
| Age mean (SD) | | 82.0 | (81.96-82.03) | 81.87 | (81.83-81.91) |  | 81.77 | (81.73-81.81) | 81.86 | (81.82-81.91) |  |
| Gender | |  |  |  |  |  |  |  |  |  |  |
|  | male | 39,633 | 22.9% | 22,094 | 25.1% | -5.2 | 22,241 | 26.1% | 21,937 | 25.8% | 0.8 |
|  | female | 133,412 | 77.1% | 65,858 | 74.9% | 5.2 | 62,951 | 73.9% | 63,255 | 74.2% | -0.8 |
| Antdementia medications use | |  |  |  |  |  |  |  |  |  |  |
|  | Donepezil | - | - | 73,261 | 83.3% | - | - | - | 71,459 | 83.9% | - |
|  | Galantamine | - | - | 18,779 | 21.4% | - | - | - | 18,149 | 21.3% | - |
|  | Rivastigmine patch | - | - | 1,669 | 1.9% | - | - | - | 1,623 | 1.9% | - |
|  | Memantine | - | - | - | - | - | - | - | - | - | - |
| Bone fractures before entry | |  |  |  |  |  |  |  |  |  |  |
|  | None | 169,445 | 97.9% | 86,055 | 97.8% | 0.5 | 83,488 | 98.0% | 83,402 | 97.9% | 0.7 |
| Osteoporosis | | 26,014 | 15.0% | 11,464 | 13.0% | 5.8 | 12,617 | 14.8% | 11,197 | 13.1% | 4.8 |
| Osteoporosis medications before entry | |  |  |  |  |  |  |  |  |  |  |
|  | Bisphosphonate | 11,208 | 6.5% | 11,634 | 13.2% | -22.8 | 10,230 | 12.0% | 9,843 | 11.6% | 1.4 |
|  | PTH | 762 | 0.4% | 1,023 | 1.2% | -8.1 | 726 | 0.9% | 844 | 1.0% | -1.4 |
|  | Dmab | 21 | 0.0% | 466 | 0.5% | -10.0 | 21 | 0.0% | 21 | 0.0% | 0.0 |
|  | Eldecalcitrol | 3,590 | 2.1% | 5,991 | 6.8% | -23.1 | 3,577 | 4.2% | 3,671 | 4.3% | -0.5 |
|  | Alfacalcidol | 8,108 | 4.7% | 6,386 | 7.3% | -10.9 | 6,548 | 7.7% | 5,974 | 7.0% | 2.6 |
|  | SERM | 4,250 | 2.5% | 3,943 | 4.5% | -11.1 | 3,499 | 4.1% | 3,488 | 4.1% | 0.1 |
| Bone mineral density testing | |  |  |  |  |  |  |  |  |  |  |
|  | DEXA | 2,386 | 1.4% | 2,383 | 2.7% | -9.4 | 1,885 | 2.2% | 1,985 | 2.3% | -0.8 |
|  | MD | 6,902 | 4.0% | 6,395 | 7.3% | -14.3 | 5,543 | 6.5% | 5,292 | 6.2% | 1.2 |
|  | US | 1,393 | 0.8% | 1,251 | 1.4% | -5.9 | 1,048 | 1.2% | 1,072 | 1.3% | -0.3 |

AD, Alzheimer’s dementia; SD, standard deviation.

**Supplementary Table 3. Baseline characteristics of AD group with memantine and without antidementia medications.**

|  |  | Without antidemenita medications (before PS matching) | | With memantine as a monotherapy (before PS matching) | | Standardized Difference | Without antidemenita medications (after PS matching) | | With memantine as a monotherapy (after PS matching) | | Standardized Difference |
| --- | --- | --- | --- | --- | --- | --- | --- | --- | --- | --- | --- |
| Total, n | N= | 173,045 |  | 11,954 |  |  | 11,907 |  | 11,907 |  |  |
| Age (years) | |  |  |  |  |  |  |  |  |  |  |
|  | 65-69 | 9,340 | 5.4% | 378 | 3.2% | 11.1 | 395 | 3.3% | 378 | 3.2% | 0.8 |
|  | 70-74 | 26,500 | 15.3% | 952 | 8.0% | 23.1 | 986 | 8.3% | 950 | 8.0% | 1.1 |
|  | 75-79 | 27,340 | 15.8% | 2,157 | 18.0% | -6.0 | 2,109 | 17.7% | 2,152 | 18.1% | -0.9 |
|  | 80-84 | 39,299 | 22.7% | 3,388 | 28.3% | -12.9 | 3,302 | 27.7% | 3,372 | 28.3% | -1.3 |
|  | 85-89 | 40,920 | 23.6% | 3,266 | 27.3% | -8.4 | 3,233 | 27.2% | 3,249 | 27.3% | -0.3 |
|  | 90-94 | 22,570 | 13.0% | 1,486 | 12.4% | 1.8 | 1,525 | 12.8% | 1,480 | 12.4% | 1.1 |
|  | 95+ | 7,076 | 4.1% | 327 | 2.7% | 7.5 | 357 | 3.0% | 326 | 2.7% | 1.6 |
| Age mean(SD) | | 82.0 | (81.96-82.03) | 82.86 | (82.74-82.98) |  | 82.88 | (82.76-83.00) | 82.85 | (82.73-82.97) | ) |
| Gender | |  |  |  |  |  |  |  |  |  |  |
|  | male | 39,633 | 22.9% | 3,227 | 27.0% | -9.5 | 3,284 | 27.6% | 3,223 | 27.1% | 1.1 |
|  | female | 133,412 | 77.1% | 8,727 | 73.0% | 9.5 | 8,623 | 72.4% | 8,684 | 72.9% | -1.1 |
| Antdementia medications use | |  |  |  |  |  |  |  |  |  |  |
|  | Donepezil | - | - | - | - | - | - | - | - | - | - |
|  | Galantamine | - | - | - | - | - | - | - | - | - | - |
|  | Rivastigmine patch | - | - | - | - | - | - | - | - | - | - |
|  | Memantine | - | - | 11,954 | 100% | - | - | - | 11,907 | 100% | - |
| Bone fractures before entry | |  |  |  |  |  |  |  |  |  |  |
|  | None | 169,445 | 97.9% | 11,604 | 97.1% | 5.4 | 11,588 | 97.3% | 11,563 | 97.1% | 1.3 |
| Osteoporosis | | 26,014 | 15.0% | 1,425 | 11.9% | 9.1 | 1,475 | 12.4% | 1,419 | 11.9% | 1.4 |
| Osteoporosis medications before entry | |  |  |  |  |  |  |  |  |  |  |
|  | Bisphosphonate | 11,208 | 6.5% | 1,329 | 11.1% | -16.4 | 1,350 | 11.3% | 1,322 | 11.1% | 0.7 |
|  | PTH | 762 | 0.4% | 128 | 1.1% | -7.3 | 112 | 0.9% | 125 | 1.0% | -1.1 |
|  | Dmab | 21 | 0.0% | 62 | 0.5% | -9.9 | 16 | 0.1% | 16 | 0.1% | 0.0 |
|  | Eldecalcitrol | 3,590 | 2.1% | 662 | 5.5% | -18.2 | 637 | 5.3% | 646 | 5.4% | -0.3 |
|  | Alfacalcidol | 8,108 | 4.7% | 748 | 6.3% | -6.9 | 760 | 6.4% | 735 | 6.2% | 0.9 |
|  | SERM | 4,250 | 2.5% | 456 | 3.8% | -7.8 | 439 | 3.7% | 454 | 3.8% | -0.7 |
| Bone mineral density testing | |  |  |  |  |  |  |  |  |  |  |
|  | DEXA | 2,386 | 1.4% | 269 | 2.3% | -6.5 | 228 | 1.9% | 260 | 2.2% | -1.9 |
|  | MD | 6,902 | 4.0% | 641 | 5.4% | -6.5 | 575 | 4.8% | 629 | 5.3% | -2.1 |
|  | US | 1,393 | 0.8% | 156 | 1.3% | -4.9 | 131 | 1.1% | 155 | 1.3% | -1.9 |

AD, Alzheimer’s dementia; SD, standard deviation.

**Supplementary Table 4. Baseline characteristics of AD group with** **multidrug therapy and without antidementia medications.**

|  |  | Without antidemenita medications (before PS matching) | | With multidrug therapy (before PS matching) | | Standardized Difference | Without antidemenita medications (after PS matching) | | With multidrug therapy (after PS matching) | | Standardized Difference |
| --- | --- | --- | --- | --- | --- | --- | --- | --- | --- | --- | --- |
| Total, n | N= | 173,045 |  | 31,707 |  |  | 31,577 |  | 31,577 |  |  |
| Age (years) | |  |  |  |  |  |  |  |  |  |  |
|  | 65-69 | 9,340 | 5.4% | 1,386 | 4.4% | 4.8 | 1,574 | 5.0% | 1,384 | 4.4% | 2.8 |
|  | 70-74 | 26,500 | 15.3% | 3,569 | 11.3% | 12.0 | 3,844 | 12.2% | 3,558 | 11.3% | 2.8 |
|  | 75-79 | 27,340 | 15.8% | 7,603 | 24.0% | -20.6 | 7,372 | 23.3% | 7,582 | 24.0% | -1.6 |
|  | 80-84 | 39,299 | 22.7% | 10,225 | 32.2% | -21.5 | 9,730 | 30.8% | 10,180 | 32.2% | -3.1 |
|  | 85-89 | 40,920 | 23.6% | 6,879 | 21.7% | 4.7 | 6,767 | 21.4% | 6,835 | 21.6% | -0.5 |
|  | 90-94 | 22,570 | 13.0% | 1,801 | 5.7% | 25.5 | 1,945 | 6.2% | 1,795 | 5.7% | 2.0 |
|  | 95+ | 7,076 | 4.1% | 244 | 0.8% | 21.7 | 345 | 1.1% | 243 | 0.8% | 3.4 |
| Age mean(SD) | | 82 | (81.96-82.03) | 81 | (80.71-80.85) |  | 81 | (80.63-80.77) | 81 | (80.71-80.84) |  |
| Gender | |  |  |  |  |  |  |  |  |  |  |
|  | male | 39,633 | 22.9% | 8,618 | 27.2% | -9.9 | 8,865 | 28.1% | 8,609 | 27.3% | 1.8 |
|  | female | 133,412 | 77.1% | 23,089 | 72.8% | 9.9 | 22,712 | 71.9% | 22,968 | 72.7% | -1.8 |
| Antdementia medications use | |  |  |  |  |  |  |  |  |  |  |
|  | Donepezil | - | - | 24,808 | 78.2% | - | - | - | 24,699 | 78.2% | - |
|  | Galantamine | - | - | 10,103 | 31.9% | - | - | - | 10,067 | 31.9% | - |
|  | Rivastigmine patch | - | - | 2,212 | 7.0% | - | - | - | 2,202 | 7.0% | - |
|  | Memantine | - | - | 31,707 | 100% | - | - | - | 31,577 | 100% | - |
| Bone fractures before entry | |  |  |  |  |  |  |  |  |  |  |
|  | None | 169,445 | 97.9% | 31,057 | 97.9% | -0.2 | 31,106 | 98.5% | 30,939 | 98.0% | 4.0 |
| Osteoporosis | | 26,014 | 15.0% | 4,465 | 14.1% | 2.7 | 4,921 | 15.6% | 4,450 | 14.1% | 4.2 |
| Osteoporosis medications before entry | |  |  |  |  |  |  |  |  |  |  |
|  | Bisphosphonate | 11,208 | 6.5% | 3,814 | 12.0% | -19.2 | 3,848 | 12.2% | 3,793 | 12.0% | 0.5 |
|  | PTH | 762 | 0.4% | 306 | 1.0% | -6.3 | 248 | 0.8% | 298 | 0.9% | -1.7 |
|  | Dmab | 21 | 0.0% | 149 | 0.5% | -9.3 | 20 | 0.1% | 20 | 0.1% | 0.0 |
|  | Eldecalcitrol | 3,590 | 2.1% | 2,050 | 6.5% | -21.8 | 1,874 | 5.9% | 1,987 | 6.3% | -1.5 |
|  | Alfacalcidol | 8,108 | 4.7% | 1,912 | 6.0% | -6.0 | 1,954 | 6.2% | 1,885 | 6.0% | 0.9 |
|  | SERM | 4,250 | 2.5% | 1,282 | 4.0% | -9.0 | 1,214 | 3.8% | 1,271 | 4.0% | -0.9 |
| Bone mineral density testing | |  |  |  |  |  |  |  |  |  |  |
|  | DEXA | 2,386 | 1.4% | 799 | 2.5% | -8.3 | 681 | 2.2% | 763 | 2.4% | -1.7 |
|  | MD | 6,902 | 4.0% | 2,042 | 6.4% | -11.0 | 1,871 | 5.9% | 2,013 | 6.4% | -1.9 |
|  | US | 1,393 | 0.8% | 438 | 1.4% | -5.5 | 355 | 1.1% | 422 | 1.3% | -1.9 |

AD, Alzheimer’s dementia; SD, standard deviation.

**Supplementary Table5. Outcomes in the AD group with ChEI, memantine, or multitherapy, and without antidementia medications after propensity score matching.**

| Group | Outcome | Without antidementia medications | With antidementia medications | Risk difference | |
| --- | --- | --- | --- | --- | --- |
|  |  |  |  | (95% CI) | *p-*value |
| ChEI  (85,192 pairs) | Hip fracture, *n* (%) | 3,483 (4.1) | 1,387 (1.6) | -2.5% | <0.001 |
|  |  |  |  | (-2.6 to -2.3) |  |
|  | Vertebral fracture, n (%) | 5, 971 (7.0) | 5,769 (6.8) | -0.2% | 0.055 |
|  |  |  |  | (-0.5 to 0.0) |  |
|  | Radius fracture, n (%) | 547 (0.6) | 841 (1.0) | 0.3% | <0.001 |
|  |  |  |  | (0.3 to 0.4) |  |
|  | All clinical fractures, n (%) | 9,422 (11.1) | 7,725 (9.1) | -2.0% | <0.001 |
|  |  |  |  | (-2.3 to -1.7) |  |
| Memantine  (11,907 pairs) | Hip fracture, *n* (%) | 476 (4.0) | 353 (3.0) | -1.0% | <0.001 |
|  |  |  |  | (-1.5 to -0.6) |  |
|  | Vertebral fracture, n (%) | 848 (7.1) | 742 (6.2) | -0.9% | 0.006 |
|  |  |  |  | (-1.5 to -0.3) |  |
|  | Radius fracture, n (%) | 64 (0.5) | 106 (0.9) | 0.4% | 0.001 |
|  |  |  |  | (0.1 to 0.6) |  |
|  | All clinical fractures, n (%) | 1,312 (11.0) | 1,146 (9.6) | -1.4% | <0.001 |
|  |  |  |  | (-2.2 to -0.4) |  |
| Multitherapy  (31,577 pairs) | Hip fracture, *n* (%) | 1,166 (3.7) | 684 (2.2) | -1.5% | <0.001 |
|  |  |  |  | (-1.8 to -1.3) |  |
|  | Vertebral fracture, n (%) | 2,206 (7.0) | 2,137 (6.8) | -0.2% | 0.278 |
|  |  |  |  | (-0.6 to 0.2) |  |
|  | Radius fracture, n (%) | 203 (0.6) | 313 (1.0) | 0.3% | <0.001 |
|  |  |  |  | (0.2 to 0.5) |  |
|  | All clinical fractures, n (%) | 3,378 (10.7) | 2,993 (9.5) | -1.2% | <0.001 |
|  |  |  |  | (-1.7 to -0.7) |  |

AD, Alzheimer’s dementia; ChEI, Acetylcholinesterase inhibitors; CI, confidence interval.
